# An Evaluation of the Accuracy of Point of Care Ultrasound by Family Physicians in Rural British Columbia, Canada: Methodological Insights

**DOI:** 10.1111/ajr.70193

**Published:** 2026-04-22

**Authors:** Anshu Parajulee, Oron Frenkel, Virginia Robinson, Patti Janssen, Jude Kornelsen

**Affiliations:** ^1^ Department of Family Practice, Faculty of Medicine University of British Columbia Vancouver Canada; ^2^ Providence Health Care Vancouver Canada; ^3^ Rural Coordination Centre of British Columbia Vancouver Canada; ^4^ School of Population and Public Health, Faculty of Medicine University of British Columbia Vancouver Canada

## Abstract

**Objective:**

This study investigated the diagnostic accuracy of point of care ultrasound (PoCUS) by rural family physicians (FPs), using consensus ‘imaging expert’ interpretation as the reference standard.

**Design:**

Cross‐sectional.

**Setting:**

British Columbia, Canada.

**Participants:**

Five rural FPs and seven specialist physicians (imaging experts).

**Methods:**

Rural FPs saved images from their PoCUS scans over 2–10 weeks. For each scan, two expert reviewers, who were unaware of the identity of FP study participants and their interpretation of scans, independently interpreted images.

**Main Outcome Measures:**

Image interpretation agreement between FPs and imaging experts: percentage agreement, sensitivity, and specificity. Inter‐rater reliability between expert reviewers when interpreting images, as measured by percentage agreement.

**Results:**

Seven imaging experts reviewed images from 55 scans performed by five rural FPs. Among the 20 scans with expert pair agreement (that was not ‘unable to assess’), expert interpretation was the same as an FP's for all but one scan (95%). For the other 35 scans, experts in a pair (a) both could not make an assessment based on images provided (‘unable to assess’) (*n* = 4) or (b) had differing interpretations (*n* = 31).

**Conclusion:**

We found interpretation agreement among imaging experts for a minority of scans, resulting in inadequate power to assess the diagnostic accuracy of PoCUS by rural FPs. When using secondary image review to assess rural PoCUS quality, we recommend limiting the number of PoCUS working diagnoses being investigated, developing image sharing guidelines for FP participants, and using consensus agreement by multiple experts as the reference standard.

## Highlights

What is already known on this subject

○ Studies from various high‐income countries have found that the accuracy of diagnostic PoCUS by FPs tends to be high, but more clinical research is needed, particularly from rural jurisdictions.
○ In rural Canada, PoCUS is commonly used by FPs in emergency departments.
○ The only clinical study from rural Canada on PoCUS quality was conducted over a decade ago and reported findings from one FP for one working diagnosis.

What this paper adds

○ Insight into the methodological challenges of using secondary image review as a reference standard to assess PoCUS quality in rural communities.
○ Methodological recommendations for obtaining adequate sample sizes when using secondary image review to determine the accuracy of diagnostic PoCUS by rural FPs.

## 1 Introduction

Point of care ultrasound (PoCUS) is used by family physicians (FPs) globally to aid with diagnosis [1]. For PoCUS to be a safe and effective health technology, FPs must have the appropriate training and experience, and their PoCUS use should be embedded in quality assurance systems [2]. The consequences of PoCUS errors can be serious, particularly in rural acute care settings where clinicians need to make critical transport decisions [3]. Overlooking a condition can delay appropriate treatment, whereas a flawed diagnosis can lead to unnecessary treatment [1].

In response to the increased use of PoCUS by FPs, more medical schools are integrating PoCUS into their curriculum [4, 5, 6, 7, 8], including in Canada, the setting of the current study. In 2018, a panel of Canadian PoCUS educational experts used a consensus process to identify 85 ultrasound elements that Canadian medical schools should consider for inclusion in their undergraduate curriculum [9]. As of 2014, half of Canadian medical schools had a focused ultrasound component in their undergraduate curriculum [8]. A study conducted in 2016 found that almost all directors of family medicine residency programs in Canada believed PoCUS should be part of family medicine residency training curricula [7]. Yet, only about one‐fifth reported having an established PoCUS component in their program. At the time of writing, the College of Family Physicians of Canada had not yet released a statement on PoCUS. The American Academy of Family Physicians, which asserts that PoCUS is especially useful for FPs in resource‐limited settings, has recommended since 2016 that family medicine residency programs include PoCUS competencies in their curricula [10].

In rural Canada, where PoCUS is commonly used in emergency departments [11, 12, 13, 14], approximately half of FPs practice emergency medicine [15, 16]. FPs in Canada can complete additional emergency medicine residency training and obtain a Certificate of Added Competence in Emergency Medicine awarded by the College of Family Physicians of Canada [17]. Kim et al. [18]. found that all training programs in Canada for this certificate include PoCUS training but vary in key components such as curricular content and competency assessment. Only about one‐third were found to have a PoCUS quality assurance process in place for trainees. After undergraduate and postgraduate medical education, FPs in Canada can access various types of PoCUS training offered by different organizations.

Two recent systematic reviews on the quality of diagnostic PoCUS by FPs found a satisfactory level of accuracy, but both reviews concluded that more research is needed [1, 19]. Only a few studies included in the two reviews were from rural jurisdictions, with each rural study focusing on a specific diagnosis or organ system. Compared to urban FPs, rural FPs tend to have a broader scope of practice and work in more resource‐limited contexts with no or limited consultative imaging options, necessitating rural‐specific PoCUS studies [19].

A study that examined all types of PoCUS applications by rural FPs in New Zealand, published after the two reviews, found that compared to definitive findings in patient charts, 10% of PoCUS findings were incorrect [20]. Charts also showed that PoCUS did not seriously negatively impact the outcome of any patient. According to the study's authors, this indicated that FPs were aware of the limits of their PoCUS skills and gave more weight to other information collected during a patient assessment.

Using PoCUS without a local formal quality assurance system and guidance from one's national professional body can be challenging. Morton et al. [21]. found a lack of quality assurance or image review to be a barrier to PoCUS use for 39% of rural clinicians using PoCUS in the Canadian province of British Columbia (BC). Additionally, lack of rural PoCUS guidelines was a barrier for 28%, and fear of litigation was a barrier for 14%. In a recent qualitative study in the same setting, FPs reported exhibiting a high degree of caution when using PoCUS, such as by limiting PoCUS to answer specific ‘yes or no’ questions and staying within the boundaries of their training, experience, and expertise, both as FPs and PoCUS users [22]. These FPs also reported using various strategies to monitor and improve their PoCUS competency, such as obtaining feedback from informal PoCUS peer support networks and initiating parallel imaging studies. FPs from Denmark who use PoCUS have reported using similar cautious approaches, including when expanding their catalogue of PoCUS applications [23].

The Canadian Association of Radiologists published a position statement on PoCUS use that included concerns for potential harm from PoCUS if physicians not specialized in imaging are not “held to the same rigorous quality standards as those conducted by an imager” [24]. In response, various Canadian medical groups published a multidisciplinary joint statement stressing that PoCUS is an imaging modality distinct from consultative ultrasound [25], which is conducted in a dedicated imaging unit or facility and is usually more detailed and comprehensive. The joint statement also pointed out that safe and effective PoCUS use has been established in many centres in Canada but did not mention the rural context. Evidence on PoCUS quality from rural Canada is scarce. The only clinical study on quality was conducted over ten years ago and reported findings for one rural FP who used PoCUS for one working diagnosis [26].

In Canada, the widespread adoption of PoCUS by FPs in rural communities, combined with the lack of family medicine specific PoCUS guidelines, makes research on the quality of PoCUS by rural FPs necessary. The primary objective of the current study was to determine the accuracy of diagnostic PoCUS by rural FPs, using consensus imaging expert interpretation of PoCUS images as the reference standard. The main outcome measures were percentage agreement between experts and FPs, and the sensitivity and specificity of FPs' PoCUS scans. The secondary objective was to measure agreement between experts when interpreting PoCUS images, as determined by the percentage of agreement.

## 2 Materials and Methods

### 2.1 Study Participants

We used a convenience sampling strategy to recruit participants from BC. We emailed rural FPs and specialist physicians in our professional networks to ask if they would like to participate in the study. Additionally, our research centre developed an FP recruitment flyer, which we posted on our website and social media. Three study partner organizations in BC distributed the flyer via email to all physicians on their email listservs.

To participate in this study, FPs needed to have at least six months of PoCUS experience after in‐person PoCUS training, use a scanner less than 10 years old, use PoCUS at least 10 times a month, and practice primarily in a community included in the BC Rural Practice Subsidiary Agreement [27]. ‘Imaging experts’ needed to be practicing in BC and be part of a specialty with standards for PoCUS/ultrasound training and use.

### 2.2 Study Protocol

We used a cross‐sectional study design. FP participants were asked to collect data on their diagnostic PoCUS scans over six weeks for a larger study that also looked at the effect of PoCUS on their clinical decision‐making. PoCUS for paediatric patients (< 18 years old) was excluded, as was PoCUS for gestational age estimation. For the current study, FPs shared ultrasound images for their PoCUS diagnoses.

For each set of images that FPs deemed adequate to aid with diagnosis, they reported whether they thought the images showed the presence or absence of the corresponding working diagnosis or whether they were uncertain (‘indeterminate’). They were informed that their images would undergo secondary review but did not receive any image sharing guidelines.

Imaging experts reviewed FPs' images and provided their assessments. Experts were given only two pieces of information about each scan: the working diagnosis and the body part that images showed. They did not know the identity of FPs or their interpretation of images. Like FPs, experts shared whether they thought each set of images showed the presence or absence of the corresponding working diagnosis, or whether they were uncertain (‘indeterminate’). Experts could also select an ‘unable to assess’ option if they did not think images were adequate to assess for a working diagnosis. The results from the expert review were not shared back with FPs.

We planned to use two expert reviewers per scan and bring in a third expert to resolve disagreements. Most PoCUS accuracy studies using secondary expert review as the reference standard have used one expert reviewer per scan. The few studies using multiple reviewers have found varying levels of inter‐rater reliability for image interpretation [28, 29, 30], indicating that if only one expert reviewer is used per scan, study findings and conclusions may differ considerably depending on the specific expert(s) participating.

We deemed secondary image review the most feasible PoCUS reference standard for the rural study setting. All data were collected on the University of British Columbia's Research Electronic Data Capture platform. The study was approved by the University of British Columbia's Clinical Research Ethics Board (ID: H21‐01412).

### 2.3 Power Calculation and Analyses

Apart from one study from rural Spain, the studies included in two systematic reviews on the quality of PoCUS by FPs have found sensitivity and specificity for diagnostic PoCUS to range from 0.71 to 1.00 [1, 19]. More than half of the studies included in these reviews assessed accuracy shortly after providing PoCUS training to FP study participants with no previous PoCUS experience. Because FPs in the current study needed a minimum level of PoCUS training, experience, and use, we hypothesized that sensitivity and specificity would be high, approximately 0.90.

We used Hess et al.'s [31] method to calculate sample sizes required for sensitivity/specificity estimates at specific margins of error. A sample size of 177 was required to achieve a 5% margin of error for a 0.90 sensitivity/specificity estimate. Smaller samples would result in higher margins of error for this estimate: 87 for a 7.5% margin of error and 54 for a 10% margin of error.

The percentage of scans with interpretation agreement between two experts was calculated. Among scans with interpretation agreement that was not ‘unable to assess’, we planned to calculate (a) the percentage of scans with interpretation agreement between GPs and experts and (b) the sensitivity and specificity of FP PoCUS findings. We decided to consider ‘indeterminate’ as not different from ‘present’ because an ‘indeterminate’ PoCUS finding indicates the working diagnosis cannot be eliminated based on images alone, and additional clinical information is needed. This approach was also used by Becker et al. [32]. in their study of PoCUS for small bowel obstruction. Analyses were conducted in SPSS Version 28.

## 3 Results

### 3.1 Study Participants

Five FP participants from five rural BC communities joined the study. These communities varied in population size, from less than 5000 to more than 20 000. All FPs used PoCUS at least once per clinical day/shift. Experience with PoCUS ranged from two years to more than 10 years. FPs, who joined the study on different dates, collected data sometime between August 2022 and February 2023.

Seven of the nine physician imaging experts we contacted accepted an invitation to participate in this study. These physicians had a specialty in cardiology, emergency medicine, internal medicine, obstetrics/gynaecology, or radiology. Each was assigned to one or more PoCUS organ systems aligned with their specialty. One expert was able to review only three of the nine scans assigned to them; the remaining six scans were assigned to another expert. The other six experts, who each reviewed all images assigned to them, reviewed between five and 47 scans. Image review occurred between June and October 2023.

### 3.2 PoCUS Scans

Altogether, FPs collected data on 68 scans. Most scans were from an emergency department (*n* = 59); the remaining were from a primary care clinic (*n* = 7) or a hospital inpatient unit (*n* = 2). The total number of days that FPs collected data ranged from five to 19, and their total scan sample size ranged from five to 25.

**FIGURE 1:**
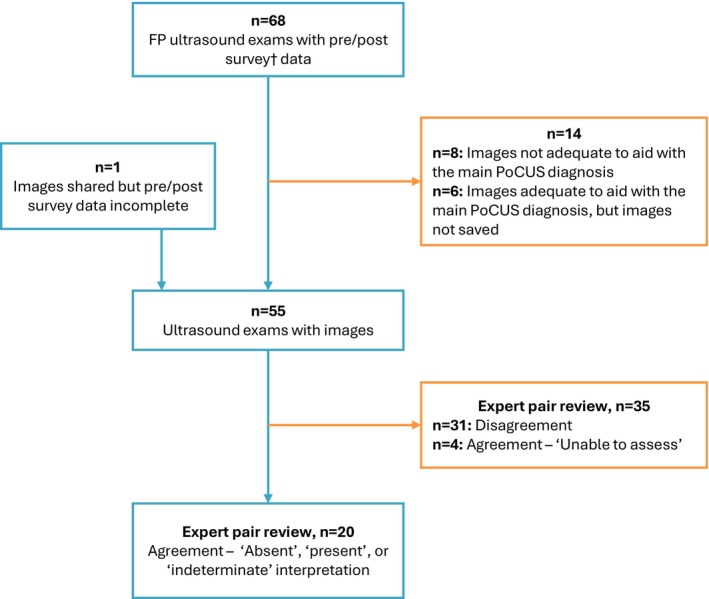
Study flow diagram. † FP participants also collected pre/post survey data on their PoCUS scans for a separate, related study on the effect of PoCUS on their clinical decision making.

**FIGURE 2:**
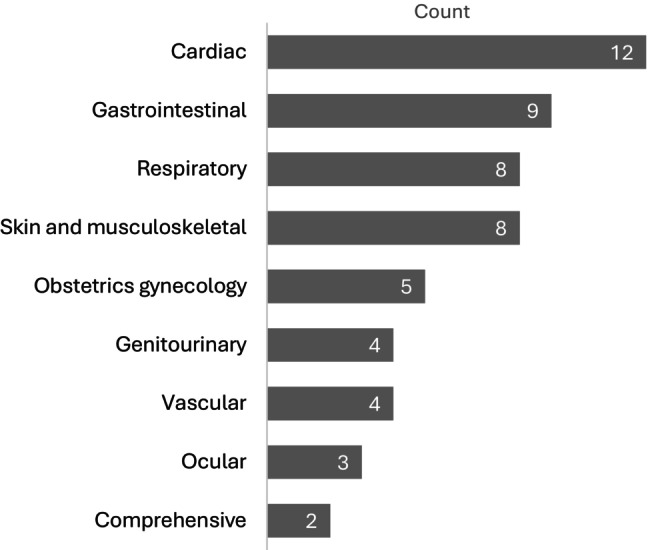
Organ systems examined by PoCUS (*n* = 55).

Fourteen scans could not be included in the current quality study, either because FPs found images inadequate to aid with diagnosis or because they did not save images (Figure 1). All nine organ systems, as defined by BC PoCUS [33], were represented in the remaining 55 scans, with cardiac scans being the most common (*n* = 12; 22%) (Figure 2). For 35 scans (64%), experts had differing interpretations or agreed that images were inadequate to assess for the corresponding PoCUS working diagnosis. Interpretation between experts and FPs could be compared for the remaining 20 scans.

### 3.3 Interpretation Agreement Between Experts

**FIGURE 3:**
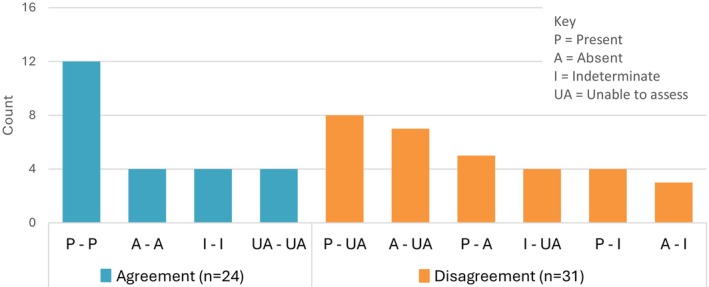
Expert review: Interpretation agreement/disagreement between experts (*n* = 55).

As there was interpretation agreement between experts for less than half of scans (*n* = 24; 44%), a third expert was not brought in to resolve disagreements. Among scans with agreement, half showed the presence of a medical condition (’present’), and one‐sixth were deemed to have an inadequate set of images (‘unable to assess’) (Figure 3). The most common type of disagreement was when (only) one expert in a pair provided an ‘unable to assess’ evaluation (*n* = 19; 61%).

**FIGURE 4:**
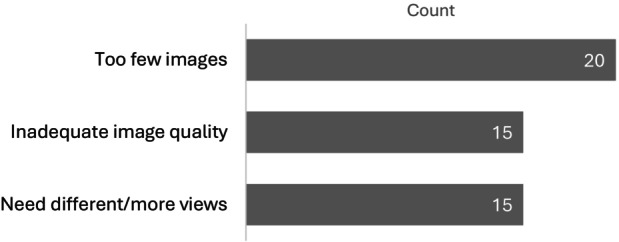
Reasons for an ‘unable to assess’ selection by experts (23 scans).

Overall, one or both experts in a pair provided an ‘unable to assess’ evaluation for 42% of scans (*n* = 23). Experts selected ‘unable to assess’ a total of 27 times and provided their rationale for their selection. The most common reason was too few images, followed by inadequate image quality and needing different/additional views (Figure 4). The number of images shared by FPs per scan ranged from one to seven. One image was shared most often (*n* = 22; 40%), followed by two (*n* = 16; 29%).

In their comments, experts identified other issues with images, including not labelling images appropriately (e.g., location on chest), not providing needed measurements (e.g., aortic measurements for abdominal aortic aneurysm), and not providing video images. Some working diagnoses were not considered appropriate PoCUS diagnoses, either because they were symptoms/syndromes rather than diagnoses (e.g., renal colic) or were diagnoses that could not be ruled in or out using ultrasound (e.g., chronic obstructive pulmonary disease). Comments also provided insight into the ways that disagreement could occur. Experts could have differing criteria for the number, type, and quality of images needed to rule in or out. Their clinical reasoning, including assumptions about other clinical data not provided, could differ. Their interpretation of images for diagnoses not considered typical PoCUS diagnoses could also differ. Table 1 outlines five instances of disagreement in which (only) one expert in a pair had a ‘present’ interpretation.

**TABLE 1:** Examples of disagreement between imaging expert reviewers when interpreting FPs' PoCUS scans.^a^

Working diagnosis	Number of images shared by FP	Expert A interpretation	Expert B interpretation	Expert comments
Congestive heart failure	4 video clips of a heart and lungs	Present	Absent	*Expert B*: Do not see any significant B lines.^b^ (No comment from Expert A)
Ectopic pregnancy	3 still images of a uterus	Present	Indeterminate	*Both experts*: Do not see a yolk sac implanted into the uterus. *Expert B*: Need additional clinical information to confirm ectopic pregnancy, e.g., positive pregnancy test, symptoms, level of human chorionic gonadotropin.
Foreign body in hand	1 still image of a hand	Present	Indeterminate	*Expert B*: There is obvious buildup indicative of an abscess, but no obvious foreign body. (No comment from Expert A)
Gallstone	1 still image of a gallbladder	Present	Unable to assess	*Expert A:* A sagittal view would have helped, but am convinced a gallstone is present based on the one still image. *Expert B*: Need more images/additional views.
Renal colic	4 video clips of kidneys	Present	Indeterminate	*Both experts*: Renal colic is a symptom or clinical presentation, not a working diagnosis. Images show the presence of hydronephrosis. *Expert A*: Images are consistent with renal colic. Assuming that the actual working diagnosis is hydronephrosis. *Expert B*: A kidney stone is not visible.

^a^ Seven imaging expert participants were assigned to scans based on their specialty. Experts ‘A' and ‘B' are not the same across scans. Summaries of comments (not verbatim comments) are shown.

^b^ More than three B lines indicate abnormally low air content in a lung [34].

### 3.4 Interpretation Agreement Between Experts and FPs

For 20 scans (36%), experts in a pair agreed that a working diagnosis was ‘present’, ‘absent’, or ‘indeterminate’. Scans were for working diagnoses across eight of the nine BC PoCUS organ systems [33]; ocular was the only organ system not represented. For all three ocular scans, at least one expert commented that high‐quality PoCUS images are challenging to obtain.

Although all five FP participants contributed at least one scan to the current study, interpretation could be compared against that of experts for only three FPs. Images for all scans by the other two FPs received an ‘unable to assess’ evaluation from at least one expert reviewer.

Of the 20 scans for which FP interpretation could be compared against that of experts, there was interpretation agreement between FPs and experts for 17 (85%) (Table 2). When ‘present’ and ‘indeterminate’ were grouped, there was agreement between FPs and experts for all but one scan (*n* = 19; 95%) (Table 2). The one scan with disagreement, for a working diagnosis of pulmonary embolism, had an absent interpretation from an FP and an indeterminate interpretation from experts. Both expert reviewers for this scan commented that images of a leg can be used to assess for deep vein thrombosis, but not pulmonary embolism, though the two diagnoses are related. There was inadequate power to assess sensitivity (*n* = 15) and specificity (*n* = 5).

**TABLE 2:** Comparison of PoCUS image interpretation between FPs and imaging experts^a^.

		FP interpretation			Total
		Absent	Present	Indeterminate	
**Consensus expert interpretation**	**Absent**	4	0	0	4
	**Present**	0	11	1	12
	**Indeterminate**	1	1	2	4
	**Total**	5	12	3	20

^a^ Colour key: Orange = disagreement; blue = agreement, same interpretation; light blue = agreement, indeterminate and present interpretations. Because indeterminate indicates that a working diagnosis cannot be ruled out and further investigation is likely needed, it is grouped with present.

## 4 Discussion

Of the 20 scans that could be assessed for accuracy using consensus expert interpretation as the reference standard, FPs and experts had the same interpretation for 19 scans (95%). There was inadequate power to precisely estimate the sensitivity and specificity of PoCUS by FP participants. The remainder of this discussion will compare the current study's methodology with that used in similar studies and explore strategies to attain adequate sample sizes.

Similar to our experience, Wordsworth and Scott [35] found it challenging to use secondary image review to determine the accuracy of rural PoCUS across a broad range of applications. In their study, which took place in rural Scotland in 1996–97, expert reviewers were not able to interpret images for 38% of scans. The authors guessed that reviewers would have been able to interpret images for more scans if video clips or a greater number of still images had been available. In the current study, we initially allowed FPs to share four still images/videos and then increased this to seven upon the request of an FP early on in data collection. Yet, for a majority of scans, only one or two images were shared. An important consideration when collecting images online is the capacity of a data collection platform to receive and store image files, which can be large.

In Nixon et al.'s [20] investigation of PoCUS in rural New Zealand in 2012, imaging experts were not able to interpret images from 8% of FPs' scans. Similarly, in our study, expert pairs agreed that images were inadequate for 7% of scans. However, for an additional 35% of scans, one expert in a pair found images to be inadequate. Like the current study, FPs in Nixon et al.'s study could collect data on any type of PoCUS scan and did not receive any guidelines for saving images. It is not clear how many expert reviewers Nixon et al. used per scan in their study.

In the current study, FPs shared images for secondary review, but for 42% of scans, experts wanted more images, additional views, and/or better image quality. This difference in perception between FPs and experts about the adequacy of images for secondary review may reflect discipline‐specific differences in standards/norms for ultrasound training and use. There may also have been facility and individual‐level factors that influenced the criteria FPs used when assessing image adequacy. Inconsistency in image interpretation among experts indicates that some of these factors may have also been relevant for this group. For instance, requiring evidence of a positive pregnancy when reviewing images for a working diagnosis of ectopic pregnancy versus assuming an FP would only try to rule out ectopic pregnancy after confirming a positive pregnancy. Our study findings also indicate that one expert reviewer per scan may not be a reliable reference standard.

The high degree of interpretation disagreement between experts in the current study reflected methodological issues, most notably the lack of image sharing guidelines for FP participants. In their study of PoCUS accuracy for hydronephrosis among emergency physicians in an emergency department, Pathan et al. [28]. used multiple expert reviewers per scan and found a high level of interpretation agreement between their two radiologist expert reviewers. Their methodology differed from ours in several key ways. In their study, only scans with a ‘complete’ set of video images were included: four clips, each at least six seconds long, that included swipe motion views of longitudinal and transverse axis views of each kidney. The kappa agreement coefficient between the two radiologists for the presence or absence of hydronephrosis was 0.77, considered to be good or very good. These two experts were from the same medical specialty and practiced in the same hospital, which may have resulted in similar image review practices. This, combined with the requirement of a ‘complete’ set of images, likely resulted in a fairly high level of interpretation agreement. In the current study, expert reviewers had various specialty backgrounds and practiced in different facilities across BC.

Studies that use expert secondary image review as the reference standard often focus on one specific PoCUS application. In urban settings with high patient volumes, an adequate sample size for one PoCUS application can be achieved from fewer providers and facilities in a shorter period. For example, the three urban hospitals that participated in Becker et al.'s [32] study of PoCUS for small bowel obstruction had a combined annual emergency department census of 250 000. The entire population of many rural communities in BC is less than 5000 [36], and rural FPs often practice in multiple clinical settings. This means that, compared to urban studies, rural studies will likely need to recruit more physicians/facilities and collect data for a longer period.

The few rural studies that have reported PoCUS accuracy for a specific working diagnosis collected data for many months. Blois, a physician‐researcher, collected data over 10 months on PoCUS scans they conducted for abdominal aortic aneurysms, achieving a sample size of 45 [26]. In Nixon et al.'s study, 28 FPs from six rural hospitals collected data on 1014 PoCUS scans over nine months. Accuracy was calculated overall across numerous PoCUS applications [20] as well as for four specific applications, with the sample size ranging from 64 to 117 for the specific applications [37, 38]. Another consideration is that rural FPs use PoCUS for a wide range of applications [13, 39, 40], some of which are more difficult and require more training, skill, and experience [1]. Thus, rural data on the diagnostic accuracy of various PoCUS applications are needed.

Providing guidelines or instructions on which images to share is a strategy used by researchers to help ensure expert reviewers can interpret images for a specific working diagnosis. For instance, Becker et al. [32]. asked study participants to use a detailed protocol when performing PoCUS scans for small bowel obstruction. Such an approach requires clinician‐researchers to identify or develop instructions for image capture. This approach would not have been feasible for the current study because FPs could enter data for any PoCUS application.

As there was considerable interpretation disagreement between experts in our study, sharing image acquisition instructions with experts may have also improved consistency in the criteria they used to judge the adequacy of images. Focusing on specific PoCUS applications would also prevent FPs from collecting data on scans for working diagnoses not considered to be PoCUS diagnoses by experts.

### 4.1 Recommendations for Researchers

Based on our experience, we make several methodological recommendations to other researchers interested in using secondary image review to assess rural PoCUS quality. We recommend convening an expert clinician panel that includes rural generalists experienced in PoCUS, as well as specialists from disciplines with standards for PoCUS/ultrasound training and use. Rural generalists help ensure that a study is grounded in the realities of rural practice, while specialists help ensure rigor in quality assessment. We recommend that a panel do the following:

1. Develop a set of criteria to select PoCUS working diagnoses to investigate. For example, the frequency of use/need in a typical rural community, the technical difficulty of a scan, and the potential impact on patient outcomes if PoCUS findings are incorrect.
2. Use the criteria to select PoCUS working diagnoses. When determining the number of diagnoses to investigate, consider the feasibility of the data collection protocol for FP participants. They will need to review and remember image sharing guidelines for all selected diagnoses.
3. For each selected working diagnosis, identify or develop guidelines for a ‘complete set’ of images to save for secondary review, e.g., number of images, specific views. These guidelines should be shared with both FP and imaging expert study participants, helping to ensure suitability of images for secondary review and consistency of review across imaging experts.
4. Determine the number of imaging experts who will review images for each scan. We recommend using two imaging experts per scan and bringing in a third expert to make a final assessment when there is disagreement. An alternative is to use three experts per scan and require agreement by at least two experts, but that would be more resource intensive.

## 5 Conclusion

We found a high level of agreement between FPs and imaging experts when interpreting PoCUS images, but this accuracy finding is inconclusive due to inadequate power. Rural PoCUS quality studies using a modified or different methodology are needed. Based on our experience, we recommend limiting the number of PoCUS working diagnoses that are investigated, developing guidelines for a complete set of images for each working diagnosis, and using consensus agreement by multiple imaging experts as the reference standard. Important considerations for studies conducted in rural communities include lower patient volumes and rural FPs tending to practice in multiple clinical settings. Compared to urban studies, a greater number of physician participants and a longer data collection period are likely needed to obtain an adequate sample size for a specific PoCUS working diagnosis.

## Author Contributions

**Oron Frenkel:** methodology, investigation, writing – review and editing. **Virginia Robinson:** methodology, investigation, writing – review and editing. **Patti Janssen:** conceptualization, methodology, writing – review and editing, supervision. **Anshu Parajulee:** conceptualization, investigation, funding acquisition, writing – original draft, methodology, visualization, writing – review and editing, formal analysis, project administration. **Jude Kornelsen:** conceptualization, funding acquisition, investigation, methodology, project administration, supervision, writing – review and editing.

## Funding

This work was supported by Canada's Digital Technology Supercluster and Mitacs.

## Ethics Statement

This study received ethical approval from the University of British Columbia's Clinical Research Ethics Board (ID: H21‐01412).

## Conflicts of Interest

The authors declare no conflicts of interest.

## Data Availability

Research data are not shared.
